# Rapid Review Summit: an overview and initiation of a research agenda

**DOI:** 10.1186/s13643-015-0111-6

**Published:** 2015-09-26

**Authors:** Julie Polisena, Chantelle Garritty, Craig A. Umscheid, Chris Kamel, Kevin Samra, Jeannette Smith, Ann Vosilla

**Affiliations:** Canadian Agency for Drugs and Technologies in Health (CADTH), 600-865 Carling Ave., Ottawa, ON K1S 5S8 Canada; School of Epidemiology, Public Health and Preventive Medicine, Faculty of Medicine, University of Ottawa, 451 Smyth Road, Ottawa, Ontario K1H 8M5 Canada; Knowledge Synthesis Group, Ottawa Hospital Research Institute, 501 Smyth Road, Ottawa, Ontario K1H 8L6 Canada; Translational Research in Biomedicine (TRIBE) Graduate Program, University of Split School of Medicine, Šoltanska 2, 21000 Split, Croatia; ECRI Institute - Penn Medicine AHRQ EPC and the Center for Evidence-Based Practice and the Perelman School of Medicine, University of Pennsylvania, Philadelphia, PA 19104 USA; Strategic Projects Branch, BC Ministry of Health, 5th floor, 1483 Douglas Street, PO BOX 9634 STN PROV GOVT, Victoria, British Columbia Canada

**Keywords:** Rapid reviews, Evidence synthesis, Health care decisions, Research agenda

## Abstract

The demand for accelerated forms of evidence synthesis is on the rise, largely in response to requests by health care decision makers for expeditious assessment and up-to-date information about health care technologies and health services and programs. As a field, rapid review evidence synthesis is marked by a tension between the strategic priority to inform health care decision-making and the scientific imperative to produce robust, high-quality research that soundly supports health policy and practice.

In early 2015, the Canadian Agency for Drugs and Technologies in Health convened a forum in partnership with the British Columbia Ministry of Health, the British Columbia Centre for Clinical Epidemiology and Evaluation, the Ottawa Hospital Research Institute, and the University of Pennsylvania. More than 150 evidence synthesis producers and end users attended the *Rapid Review Summit: Then, Now and in the Future*. The Summit program focused on the evolving role and practices of rapid reviews to support informed health care policy and clinical decision-making, including the uptake and use of health technology assessment.

Our discussion paper highlights the important discussions that occurred during the Rapid Review Summit. It focuses on the initial development of a research agenda that resulted from the Summit presentations and discussions. The research topics centered on three key areas of interest: (1) how to conduct a rapid review; (2) investigating the validity and utility of rapid reviews; and (3) how to improve access to rapid reviews*.*

## Background

The demand for accelerated forms of evidence synthesis—referred to in this paper as rapid reviews—is on the rise, largely in response to requests for prompt assessment and up-to-date information by health care decision makers about health care technologies, and health services and programs [1–4]. Heightened interest in rapid reviews, coupled by the dearth of published evidence, has been underscored by a recent *Systematic Reviews* journal series on the subject, accessed more than 9000 times since January 2015 [4]. Another manuscript on rapid review approaches continues to be one of the most highly accessed articles in the journal, retrieved more than 23,000 times since its publication in 2012, 800 times alone in January 2015 [5].

Three 2015 publications presented the processes and methods involved with the production of rapid reviews [2–4]. One paper explored the methods and context for the production of 36 rapid reviews from 20 organizations and conducted interviews with rapid review producers, and another study performed a descriptive analysis of processes and methods for 29 international rapid review programs [2, 3]. Both studies indicated that there is no standard definition of rapid reviews or methods to produce them, possibly attributed to the timeframe and type of evidence synthesis employed to complete a report [2, 3]. The third study proposed general principles to produce rapid reviews that were aligned with the methods used for systematic reviews to improve their transparency [4]. Rapid review evidence synthesis is marked by a tension between the strategic priority of informing health care decision-making, while addressing the scientific imperative for the production of robust, high-quality research that soundly supports health policy and practice [5, 6]. As the demand for rapid reviews continues to grow, there is an opportunity, considerable interest, and urgency for harnessing producer and end-user insights to guide the development of this field.

In early 2015, the Canadian Agency for Drugs and Technologies in Health (CADTH) convened a forum in partnership with the British Columbia Ministry of Health, the British Columbia Centre for Clinical Epidemiology and Evaluation, the Ottawa Hospital Research Institute, and the University of Pennsylvania. The *Rapid Review Summit: Then, Now and in the Future* focused on the evolving role and practices of rapid reviews to support informed health care policy and clinical decision-making, including the uptake and use of health technology assessment (HTA). More than 150 participants attended the Summit. They were primarily rapid review producers (who are among the champions of creative efforts in this area) and end users of rapid reviews (decision makers at the forefront of clinical and policy decisions). Participants came from a wide range of health research and practice settings across Canada and internationally including government, research institutions, academia, not-for-profit organizations, and industry.

A planning committee of experts in the field developed the scientific program for the Summit and articulated four objectives. They were as follows: (i) to share information among health care decision makers and providers, rapid review producers, and representatives from organizations interested in rapid reviews; (ii) to facilitate discussions concerning the production of rapid review reports and their use to support informed decision-making; (iii) to initiate the development of a priority research agenda to further advance the science of rapid reviews; and (iv) to contribute to the ongoing development of a community of practice for rapid reviews to facilitate continued information sharing and collaborations among rapid review producers and health decision makers.

This discussion paper highlights the important discussions that occurred during the Rapid Review Summit. In particular, this paper focuses on the initial development of a research agenda from the Summit presentations and discussions. The purpose of this research agenda is to identify research topics to advance the science in rapid review definitions and methods and to enhance their application to support informed health care decision-making for both the users and producers of rapid reviews.

## Methods

The scientific program was comprised of six oral sessions presented by 14 North American and international speakers. They included Jesmin Antony, Vivian Coates, Chantelle Garritty, Jeanne-Marie Guise, Chris Kamel, Janet Joy, Craig Mitton, David Moher, Michelle Mujoomdar, Susan L. Norris, Julie Polisena, Kevin Samra, Sharon E. Straus, Lesley Stewart, Andrea C. Tricco, and Craig A. Umscheid [7]. Each session lasted between 60 and 90 min. Four presenters represented decision makers, who discussed how they incorporate rapid reviews in their decision-making process and the expectations, appropriateness, and risks associated with the use of rapid reviews. Some of the other sessions presented by rapid review producers compared rapid reviews with systematic reviews, the risks and opportunities with publishing rapid reviews, and the impact of rapid reviews in HTA.

The Summit concluded with an exercise, where ideas for an ongoing research agenda were solicited from participants. The presentations and discussions were audio-recorded, and each group submitted their research ideas in writing. The detailed program is available on the CADTH website [7].

In the exercise, 11 randomly formed groups of four to six participants worked together for 40 min to propose research project ideas. They were asked to start the discussion by sharing ideas for “a priority research agenda for rapid reviews” based on their experience, expertise, and what they heard during the Summit; to identify similarities and overlaps; and then to submit the group’s top three ideas for review by the Summit planning team. Some groups submitted more than three ideas resulting in a total of 50 ideas when collated.

Following the Summit, all ideas submitted were listed verbatim and reviewed to identify unique themes in an inductive manner by one individual (JW) and verified by another reviewer (JP). A list of themes was reviewed by comparing and contrasting the ideas in each theme and to decide whether they should be expanded or merged. Two individuals (JW and JP) compared their results and achieved consensus through discussion. Unique themes that emerged from the research ideas submitted by the Summit participants helped to inform the development of a rapid review research agenda.

## Results

The Summit focused on generating dialogue in response to questions that are familiar to those most active in the evidence synthesis field, including:

- When is it appropriate to undertake a rapid review?
- What are the role of, scope of, and approaches to conducting rapid reviews?
- What potential risks of bias are associated with rapid reviews given the existing methodological approaches?
- Are rapid reviews a natural extension of systematic reviews? Should they be?
- Are they effectively influencing end-user decision-making?
- Can rapid reviews be considered credible given the lack of consistency in terminology and practice?

Seven topic areas for a research agenda emerged from the final Summit exercise, including theory and taxonomy, methods and application, comparing and contrasting with systematic reviews, evaluation of use, database development, influence on practice, and suggestions for tool and guideline development (Table 1).

**Table 1:** A research agenda for rapid reviews

Themes	Subthemes
Taxonomy and definitions	a. How the various types of RRs are defined?
	b. What are the standard definitions for rapid reviews?
	c. What are the purpose and use of the various types of RRs?
	• Understand how they support informed health care decision-making
	• Evaluate the scope and limitations of rapid reviews in health policy decision-making
Methods, process, and application	a. What are the core elements for RR development (for example, timelines, number of reviewers, number of databases searched)?
	b. What are the steps to conduct RRs?
	c. How are RR questions developed?
	• Identify and develop products that work for various review questions and contexts
	d. What trade-offs of time, resources, and comprehensiveness in various RR products impact results and conclusions?
	• Assess potential biases of RRs and the implications for reporting results
	e. What are the appropriate search strategies, including different approaches to text mining, and streamlined approaches?
	f. What are the best practices for data synthesis, reporting, and interpretation?
	g. What are the best practices for communicating results and knowledge mobilization?
	h. Is it appropriate to use RRs for health care decision-making?
	• Determine when and when not to use RR
	i. What are the best practices for working with end users?
Compare and contrast rapid reviews with systematic reviews	a. What are the similarities and differences in methods between RRs and SRs and their implications for results?
	• Investigate how to quantify bias in conclusions and recommendations
	b. What are the qualitative versus quantitative methods for RRs with SR counterparts?
	c. What are the strengths and limitations of RRs versus SRs?
	• Address potential biases, accuracy, and precision of RRs vs SRs
	• Consider perspectives of producers and users
	• Identify shortcuts that can be used in RR products without compromising their quality
Evaluate use (including for quality assurance and impact)	a. How are RRs and SRs used by various organizations?
	• Identify who are the users of RRs and how they use them
	• Measure and quantify their use and impact in health care decision-making
	b. What are the risks decision makers are willing to accept in using RRs?
	c. What is the impact on policy decisions and practice change?
	d. Is it appropriate to use rapid reviews in cost-effectiveness analyses?
Database	a. What are the opportunities and constraints for a RR international database or clearinghouse?
	b.How can the identification of RRs be improved to reduce duplicate requests?
Influencing RR practice	a. How can the application of RRs be enhanced among their users?
	• Educate end users regarding what is feasible with RRs
	• Determine what end-user need(s) RRs can meet
	• Work with users in defining the question(s), conducting the reviews, interpreting results
RR tools and guideline development	a. What tools and guidelines can be developed to improve the production and reporting of RRs?
	• AMSTAR-like tool to guide the RR process
	• Process map or decision tree to guide use of RRs and/or SRs
	• Guideline or checklist of what to include in RRs
	• Checklists that assist with selection of RR products suitable for questions
	• Flowchart of questions to support question development
	• Quality assessment of RR products based on content and validity
	• Reporting guidelines for RRs

*AMSTAR* Assessing the Methodological Quality of Systematic Reviews, *RR* rapid review, *SR* systematic review

### Taxonomy and definitions

The imperative to establish rapid review taxonomy and definitions reinforced the importance of moving forward with standard definitions, shared terminology, and concepts as a foundation for the field. Participants noted the difficulty of engaging in scientific discourse in the absence of a consistent lexicon of terms.

### Methods, processes, and application

The majority of ideas for future research focused on the area of methods and their application to guide rigorous scientific practice at every stage of rapid review product development. Suggestions included exploring methods used for problem delineation, the development of questions that are suitable in focus and scope for rapid reviews, or as one group stated, developing a “process map” for what would trigger the need for a full systematic review versus a rapid review. Research that informs best practices for accelerated evidence synthesis along with methodological guidelines was suggested. The need to describe trade-offs between time, resources, and comprehensiveness was identified to ensure that users are aware of product limitations and “the need to interpret results and conclusions with caution.”

### Comparing and contrasting with systematic reviews

Systematic reviews were the main reference point in discussions about the development of rapid review methods and practice; thus, a number of research ideas focused on the potential benefit of comparative work. One idea suggested research on the unique dynamics of the producer and end-user relationship in the development of rapid reviews. Specific reference was made to exploring the dynamics through “a comparative analysis that focuses on the perspectives of end users.” Other suggested areas of comparative study included seeking approaches to mitigate risks associated with bias and evaluating comparative levels of accuracy and uptake in health policy and practice decisions.

### Evaluation of use

Participants identified the need for rapid review evaluations to describe users, to understand how and when rapid reviews are being used, and to delineate existing practices and their outcomes. The congruence of conclusions between systematic reviews and rapid reviews, undertaken in the same timeframe on the same topics if additional resources are allocated to conducting the systematic review, was of interest to participants. With reference to product impact, an evaluation of how rapid reviews influence decision-making in health policy and practice was suggested. Interest was also expressed in reviewing policy decisions made using rapid reviews once systematic reviews have been published on the same topic. Evaluation is also needed to guide development of metrics to assist with measuring and quantifying the various dimensions of rapid reviews, including their quality and validity of content.

### Development of a database

A number of ideas focused on the importance of developing a free, international database or repository of rapid review products, as well as related tools and other resources. Development of a rapid review database reinforces the urgency for standard definitions, shared terminology, and concepts in order to ensure consistency in their categorization. Otherwise, there would be risks of jeopardizing the credibility of rapid review science. Existing practices for the registration of systematic reviews might be useful in guiding further research into logistics of database development, as well as architecture, management, opportunities, and constraints.

### Tools and guidelines

There was a high level of interest in the generation and dissemination of tools and guidelines to enhance methodological consistency and stimulate rapid review development processes. A number of agencies shared information about tools and guidelines already in use to support and systematize their rapid review development. Linked with tools and resources were suggestions for flowcharts to guide process, and product templates and guidelines to support consistency and uniformity of methodological practice. A tool that assists with “grading” rapid reviews and their level of uncertainty might be useful to decision makers. While it was noted that the lack of shared terminology and methods forms a barrier to common tools and guidelines, their development and dissemination through publication may support methodological consistency with time.

### Influencing practice

The area of influencing practice emerged as a minor yet cross-cutting theme. Reference was made to engendering collaboration, momentum, and communication of best practices to collectively advance the field. The development of a community of practice that involves end users and other key stakeholders, such as patients and the public, was cited.

## Discussion

The dialogue at the Summit highlighted the growing relevance, practices, and gaps central to rapid reviews. The research topics that emerged from the invited speakers and discussions centered on three key areas including (1) how to conduct a rapid review (i.e., deriving a taxonomy of rapid reviews, understanding their methods, application and influence on practice, suggestions for tool and guideline development); (2) how to understand validity and utility of rapid reviews (i.e., theory and taxonomy, comparing and contrasting with systematic reviews, evaluation of use); and (3) how to better access rapid reviews (i.e., developing a database or repository of rapid reviews). The authors acknowledge that several of these topics currently are being addressed or planned for in the near future.

Summit participants highlighted the priority of developing definitions and bringing more precision to terminology that describe different accelerated synthesis products. They considered this step to be essential to ongoing development, comparative research, and evaluation of rapid review use and impact. In addition, work is needed to clarify appropriate methods for all stages of product development [1–12], including the development of norms and practices to address the lack of transparency in reporting. Resources and tools are needed to support methodological decisions by outlining essential steps given different timelines, potential complexities, and type of advice being sought.

Participants expressed an interest in the development of methods for rapid reviews that benefit from and are anchored in the science of systematic reviews. Arguably, the systematic review approach has come a long way over the last 22 years and continues to be refined [13]; thus, there are substantial methods with much that can be learned from comparative studies. This area of needed research includes evaluations on the impact of streamlining systematic review methods, and comparative research to strengthen the caliber of literature searches. The purpose of the research is to explore quality assurance as it relates to the reduced number of internal reviewers with rapid reviews, to determine methods to assess and reduce the risk of bias, and to evaluate the relative impacts of these various approaches.

Good scientific endeavor in rapid reviews is built around transparency and accountability [14]. Yet rapid reviews are frequently not published given the service nature of these products. Consistent reporting, the ability to register with a central site, and publishing, either formally through peer-reviewed journals or informally using websites, social media, or other means, are needed as the science advances. The development of a centrally located repository or rapid review database was widely supported with the provision that it be guided by standard definitions, shared terminology, and concepts and categorized within the database. Questions related to the type of infrastructure, proprietary considerations, database management, and current terminology inconsistencies were highlighted for further investigation and discussion.

Numerous agencies are in the process of defining and refining their approaches to rapid reviews, creating opportunities for sharing best practices and lessons learned, and collaborating in the development of the theory, taxonomy, and methodology to guide the development and use of rapid reviews. The formation of the Cochrane Rapid Review Methods group, which proposes to provide training and support, track research, and serve as a discussion forum for rapid reviews, and the recently established Guidelines International Network (G-I-N) working group that will work toward guideline development, production, testing, and dissemination of essential materials were among the many initiatives highlighted. Forging a community of practice, composed of knowledge producers and end users, is essential for the sharing of emerging practices, lessons learned, and priority areas for further research and development [15]. The Cochrane Methods Rapid Reviews and G-I-N are promising initiatives for the growing field, given the connectivity of these organizations to the wider evidence synthesis community.

## Conclusions

Rapid reviews have become ubiquitous in health care, with local and global agencies emerging as expert hubs. Producers and end users of rapid reviews that assembled for the Summit shared focused considerations of the current state of rapid reviews, noting tremendous heterogeneity in methods, approaches, and products. The Summit formed a valuable platform for discussion of efforts currently underway to advance the science and strengthen methodologies, accountability, and transparency of rapid reviews. It also accessed the experiences and perspectives of decision makers, who spoke of contextual realities, along with tensions between the evidence base and the experience base of health care professionals, reinforcing the importance of high-level leadership that embraces the pursuit and advancement of evidence-informed policy and practice.

The research agenda generated by Summit participants aligns with the conclusions found in two 2015 studies on rapid reviews and offers a reference point and potential road map for evidence synthesis producers and end users. Yet, the importance of strategic leadership to build and promote scientific momentum was seen as essential. Constructively harnessing the talents of producers and users of rapid reviews will be instrumental in establishing and bringing scientific rigor to the field. The speakers’ presentations from the Summit are also available on the CADTH website at https://www.cadth.ca/cadth-summit-series.

## Abbreviations

AMSTAR: Assessing the Methodological Quality of Systematic Reviews
CADTH: Canadian Agency for Drugs and Technologies in Health
G-I-N: Guidelines International Network
HTA: health technology assessment
RR: rapid reviews
SR: systematic reviews
